# Body Image Satisfaction, Eating Attitudes and Perceptions of Female Body Silhouettes in Rural South African Adolescents

**DOI:** 10.1371/journal.pone.0154784

**Published:** 2016-05-12

**Authors:** Titilola M. Pedro, Lisa K. Micklesfield, Kathleen Kahn, Stephen M. Tollman, John M. Pettifor, Shane A. Norris

**Affiliations:** 1 MRC/Wits Developmental Pathways for Health Research Unit,Department of Paediatrics,/Faculty of Health Sciences, University of the Witwatersrand, Johannesburg, Gauteng, South Africa; 2 MRC/Wits Rural Public Health and Health Transitions Research Unit (Agincourt),School of Public Health,/Faculty of Health Sciences, University of the Witwatersrand, Johannesburg, Gauteng, South Africa; Newcastle University, UNITED KINGDOM

## Abstract

This study aims to examine the associations between BMI, disordered eating attitude, body dissatisfaction in female adolescents, and descriptive attributes assigned to silhouettes of varying sizes in male and female adolescents, aged 11 to 15, in rural South Africa. Height and weight were measured to determine BMI. Age and sex-specific cut-offs for underweight and overweight/obesity were determined using the International Obesity Task Force cut-offs. Body image satisfaction using Feel-Ideal Discrepancy (FID) scores, Eating Attitudes Test-26 (EAT-26), and perceptual female silhouettes were collected through self-administered questionnaires in 385 adolescents from the Agincourt Health and Socio-Demographic Surveillance System (HSDSS). Participants self-reported their Tanner pubertal stage and were classified as early pubertal (< = Tanner stage 2), and mid to post pubertal (Tanner stage > 2). Mid to post pubertal boys and girls were significantly heavier, taller, and had higher BMI values than their early pubertal counterparts (all p<0.001). The prevalence of overweight and obesity was higher in the girls than the boys in both pubertal stages. The majority (83.5%) of the girls demonstrated body dissatisfaction (a desire to be thinner or fatter). The girls who wanted to be fatter had a significantly higher BMI than the girls who wanted to be thinner (p<0.001). There were no differences in EAT-26 scores between pubertal groups, within the same sex, and between boys and girls within the two pubertal groups. The majority of the boys and the girls in both pubertal groups perceived the underweight silhouettes to be “unhappy” and “weak” and the majority of girls in both pubertal groups perceived the normal silhouettes to be the “best”. These findings suggest a need for policy intervention that will address a healthy body size among South African adolescents.

## Introduction

Adolescence, a period of transition from childhood to adulthood, is characterised by psychological, physical and social changes [1]. Considerable research has been conducted on body image, eating disorders and other weight-related behaviours among adolescent girls and young women living in developed countries [2–4], with few research studies being done in low-to-middle income countries [5–8]. The decreasing age at which body image dissatisfaction and disordered eating attitudes occur in adolescence is of public health concern globally [9–11], due to physiological and psychological health problems [12].

It is widely accepted that girls are at greater risk of body dissatisfaction and eating disorders [2], and that both a low and a high body mass index (BMI) have been shown to influence weight control behaviours [5, 13, 14]. Underweight boys who experience muscle dysmorphia may engage in weight control behaviours to gain weight and increase muscle mass [14] while overweight girls engage in other behaviours to lose weight, both leading to the development of eating disorders [12]. In addition, body dissatisfaction has been shown to be strongly related to societal norms, culture and ethnicity [14]. Increasing globalisation and exposure to a ‘Western’ ideal of thinness through the media [2, 10], which often differs from traditional beliefs, creates even greater conflict within the adolescent.

Traditionally, a larger figure has been considered desirable in African culture, as it represents wealth, beauty, and the absence of illness [15–17], however, recent studies have reported an increasing risk of eating disorders in urban Black South African children and adolescents [15, 18, 19]. Evidence from South Africa is conflicting as some studies have reported an increasing number of urban South African adolescents with a desire to be thin [7, 11], while Puoane et al. [7] have shown that among 10–19 year old Black girls living in Cape Town, there is still a desire for a higher BMI.

Few studies [8, 20, 21] have been conducted on eating attitudes and body image in adolescents from South African rural communities who have recently been shown to be at high risk of overweight and obesity [22, 23], the prevalence of which is expected to increase as these communities continue along the nutrition transition [24]. In this study, we aim to examine BMI, disordered eating attitudes, female body dissatisfaction and descriptive attributes assigned to silhouettes of varying sizes, amongst male and female adolescents in rural South Africa.

## Methods

### Sample and study setting

Data for this study were collected in rural villages in the Agincourt sub-district, located in the north-eastern part of South Africa, close to the border with Mozambique. The Agincourt Health and Socio-Demographic Surveillance System (HSDSS) was established in 1992 and collects prospective data on the community living in the Agincourt sub-district of Mpumalanga Province, South Africa. The land is subdivided into plots too small to support subsistence farming and includes 27 villages in total, with 90 000 people and 20 000 households [22, 25, 26]. Further, despite current government initiatives, infrastructure in the area is still limited and the level of income is generally low by South African standards (73% of households earn less than ZAR 9600 per annum) [22, 25].

In 2009 [26], participants (n = 600; age range 7 to 15 years (200 subjects in each group)), were randomly selected from 3489 Black South African children and adolescents (aged 1- to 20 years), who had participated in a 2007 growth survey [22]. Analysis for this study were limited to participants between 11- and 15 years of age (n = 385, 190 boys and 195 girls). Data for the 7/8 year old age group were not included due to their poor comprehension and understanding of the questionnaires. Ethical approval was granted by the University of the Witwatersrand Committee for Research on Human Subjects (Medical, M090212) and from the Mpumalanga Provincial Government Department of Health. Permission from the community leaders and schools principals, and written parental consent were obtained and assent was given by all participants. Interviews were conducted in participant’s local language by carefully trained staff at designated schools.

### Measures

Height was measured using a stadiometer (Holtain, UK), calibrated in centimetres. Participants were measured without shoes, but with light socks, in a standing position with their hands parallel to their bodies and with the back of the head touching the vertical board of the stadiometer. The participants were weighed to the nearest 0.1kg, wearing light clothing and without shoes, using a mechanical bathroom scale (Hanson; Bathroom Trends Limited, Epson, UK). BMI was calculated as weight in kilograms (kg) divided by the height in meters squared (m^2^) and underweight, normal weight and overweight and obesity were determined using International Obesity Task Force age and sex specific BMI cut-off points [27]. Pubertal assessment was measured using the Tanner 5-point pubertal self rating scale, which has been validated previously in Black South Africans [28]. Breast development in girls and genital development in boys were assessed and used to classify participants as early pubertal (≤Tanner stage 2), and mid to post pubertal (Tanner stage > 2) [28].

Body image satisfaction was measured using Stunkard’s silhouettes, which have previously been validated in South African adolescent females [16]. These silhouettes were presented randomly with each sheet of paper containing one image. From the eight body silhouettes presented to them, each female participant was required to select a body silhouette representing their current body shape (feel figure), and the body shape that they desire (ideal figure). The body silhouettes were coded 1 to 8 (from the thinnest to the fattest). Body image dissatisfaction was determined using the Feel-Ideal Discrepancy (FID) scores and which was calculated by subtracting the number of the ideal body silhouette from the feel body silhouette. Zero, positive and negative scores indicated contentment with body shape, desire to be thinner, and desire to be fatter, respectively [16, 17].

Further, the 8 silhouettes were randomly placed on a table and each of the participants (boys and girls) were asked to associate different body shapes from different silhouettes presented to them. Descriptions included the “best, worst, clumsy, has more respect, has less respect, the strongest, the weakest, the happiest and unhappy”. The silhouettes were coded from 1 (thinnest) to 8 (biggest) and then grouped into 4 categories; silhouettes 1 and 2 (underweight), 3 and 4 (normal), 5 and 6 (overweight) and 7 and 8 (obese) [5].

The 26-item Eating Attitudes Test (EAT-26) was used to evaluate the adolescents’ risk of a future eating disorder [29]. The EAT-26 has been widely used and has been shown to be valid in both rural and urban South African children [20, 30]. EAT-26 consists of 26-items scored on a Likert scale (0—never to 6 -always) and the items are summed to obtain an overall score (range = 0–78). Adolescents with an overall score 20 are considered to be at risk of developing a future eating disorder. The three sub-scores of the EAT-26 were used to evaluate dieting, bulimia and food preoccupation, and oral control. The internal consistency (Cronbach Alpha) of the EAT-26 questionnaire and EAT-26 sub-components were low (0.50–0.60). Further examination revealed that some questionnaire items may have been poorly understood. In particular: (i) I am terrified about being overweight; (ii) I avoid eating when I am hungry; (iii) I am aware of the calorie content of foods that I eat; (iv) I engage in dieting behaviour.

### Data analysis

All the statistical analysis were completed using STATA version 11 (StataCorp, Texas, USA). Using Student *t*-tests, differences between pubertal stage of development and sex were computed for normally distributed data (weight, height and BMI). The results were expressed as mean ± standard deviation (SD). The Wilcoxon-Mann-Whitney test was used to determine significant differences by sex and by pubertal stages for EAT-26 and EAT-26 sub-scores which were not normally distributed and the median and inter-quartile ranges (IQR) were reported. A one-way analysis of variance (ANOVA) test was used to evaluate if there were significant differences in BMI between body image dissatisfaction groups, with the post-hoc Bonferroni test to determine where significant differences exist. Pearson’s chi-squared tests were used to determine significant differences between categorical variables (i.e. EAT-26 scores < or ≥ 20, BMI and FID categories, and perceptual female silhouettes) between pubertal groups, and boys and girls and the percentages (%) were reported. The significance level was set at P<0.05.

## Results

The descriptive characteristics of the participants by sex and pubertal stage of development are presented in Table 1. A total of 44.7% participants were classified as early pubertal (12.4±1.1 years of age) and 55.3% participants (14.5±1.3 years of age) were classified as mid to post pubertal. Within the early pubertal group, boys were significantly older than the girls in the same pubertal group, but there were no significant differences in weight, height or BMI between boys and girls in the early pubertal stage. Within the mid to post pubertal group, there was no significant difference in age, however, the girls were significantly heavier and shorter and had a higher BMI than the boys. Mid to post pubertal boys and girls were significantly older, heavier, taller, and had a higher BMI than their early pubertal counterparts (all p<0.001).

**Table 1:** Subjects characteristics by sex and pubertal stages of development.

Variables	Early pubertal stage (Tanner stages 1 & 2)		Significant differences by sex	Mid to post pubertal stage (Tanner stages > 2)		Significant differences by sex	Significant differences by pubertal stages (A & B)
	Boys (N = 99)	Girls (N = 73)		Boys (N = 91)	Girls (N = 122)		
**Age (years)**	12.6±1.2	12.2±1.0	**0.024**	14.5±1.4	14.4±1.3	0.547	
**Weight (kg)**	36.4±7.1	38.7±1.0	0.074	49.4±11.1	53.3±12.0	**0.016**	**0.001**^A^,^B^
**Height (cm)**	146.0±8.1	147.5±8.0	0.203	161.4±10.1	158.1±6.4	**0.004**	**0.001**^A^,^B^
**BMI (kg/m^2^)**^a^	17.0±2.0	17.5±3.3	0.198	19.0±2.6	21.2±4.0	**0.001**	**0.001**^A^,^B^
*EAT 26 scores	10 (6–15)	12 (6–17)	0.180	12 (9–15)	10 (6–14)	0.067	0.833^A^, 0.833^B^
*EAT 26 subcomponents scores	**(N = 95)**	**(N = 66)**		**(N = 89)**	**(N = 112)**		
Dieting	4.8 (2–7)	4.9 (3–6)	0.871	5.5 (3–7)	5.1 (3–7)	0.174	0.516^A^, 0.385^B^
Bulimia and food preoccupation	2.4 (0–4)	2.0 (0–3)	0.424	2.4 (0–3)	1.8 (0–3)	0.064	0.618^A^, 0.533^B^
Oral control	3.4 (0–6)	4.7 (1–7)	**0.024**	3.9 (1–6)	3.4 (1–6)	0.015	0.921^A^, 0.115^B^
**BMI category**							
Underweight	4(4.0%)	7(9.6%)	0.01 (*χ*^2^ = 8.802)	6(6.6%)	4(3.3%)	0.001 (*χ*^2^ = 13.332)	0.452^A^, 0.179^B^
Normal weight	93(94.0%)	58(79.3%)		82(90.1%)	94(77.1%)		
Overweight and obesed combined	2(2.0%)	8(11.1%)		3(3.3%)	24(19.7%)		
*EAT-26 Cut-offs							
EAT-26 score < 20	85(89.5%)	59(89.4%)	0.978	82(92.1%)	103(92.0%)	0.965	0.701^A^, 0.547^B^
EAT-26 score ≥ 20	10(10.5%)	7(10.6%)		7(7.1%)	9(8.0%)		

All values are presented as means ± standard deviation (SD) for normally distributed data (Student *t* test), median IQR reported for non normally distributed data and n(%) for categorical data. Comparison by sex and pubertal stages were completed using the Wilcoxon-Mann-Whitney test (median, IQR reported). *χ*^2^-tests were used to determine significant differences for BMI and EAT-26 cut-offs categories.

^A^—Significant differences between early and mid-post pubertal stage in boys.

^B^—Significant differences between early and mid-post pubertal stage in girls.

*Variation in sample size due to missing information.

^*a*^IOTF age and gender specific BMI cut-offs for age 12.5 and 14.5 years adolescent boys and girls [27].

In Table 1, the percentage distribution of participants in the BMI categories of underweight, normal weight, and overweight/obese are shown for the boys and girls in the two pubertal groups. Although there were significant differences between the boys and girls categorised as underweight, normal weight and overweight/obese in both pubertal groups, there were no differences between the pubertal groups.

**Fig 1:**
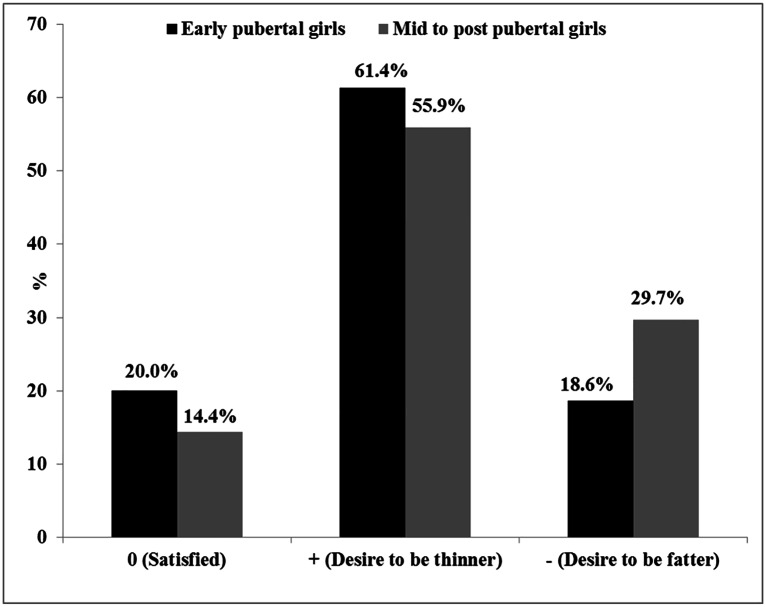
Female adolescents’ body image dissatisfaction.

For the total sample of girls (n = 188), the majority (58%) had a positive FID score, which is associated with a desire to be thinner, while 25.5% had a negative FID score, which is associated with a desire to be fatter, and 16.5% were satisfied with their bodies. The majority (83.5%) of the girls demonstrated body dissatisfaction. Within the early pubertal girls, 20% were satisfied with their current body size, while 18.6% had a negative FID score and 61.4% had a positive score. The majority 85.6% of mid to post pubertal girls were dissatisfied with their body size (55.9% had a positive FID score and 29.7% had negative FID score) and 14.4% were satisfied with their bodies. There was no difference between the pubertal groups for FID category (Fig 1).

Examination of the relationship between BMI and body dissatisfaction is presented in Table 2. There was a consistent trend, where girls in both pubertal groups who had negative FID score (desired to be fatter) had the highest body mass index and girls who desired to be thinner had the lowest body mass index in their respective pubertal groups. There was a significant difference in BMI between participants who desired to be fatter, and those who were satisfied with their current body sizes, and the girls who wished to be thinner (all p<0.001) (Table 2).

**Table 2:** Difference in BMI between the FID categories.

	Early-Pubertal Girls (n = 70)	Mid to Post-Pubertal Girls (n = 118)	P-value
	Mean±sd	Mean±sd	
**Desire to be fatter**	20.41±4.1 [n = 13]	23.81±4.9 [n = 35]	0.001^C^
**Satisfied**	17.35±1.7 [n = 14]	21.04±2.7 [n = 17]	
**Desire to be thinner**	16.68±3.0 [n = 43]	19.89±3.1 [n = 66]	0.001^D^

Values are presented as means ± standard deviation (SD) using ANOVA (one-way).

^C^—Significant differences in BMI between girls who desired to be fatter and those girls who are satisfied with their current body sizes.

^D^—Significant differences in BMI between girls who desired to be thinner and those girls who are satisfied with their current body sizes.

The difference in FID scores which represent the feel and ideal body image and the FID score chosen by early and mid to post pubertal girls are presented in (Table 3). The silhouettes chosen by the early pubertal girls to represent the ideal body image had a significantly higher score than that chosen by the mid to post pubertal girls (p = 0.045). There was no difference in the chosen feel body image or the FID scores between the pubertal groups. The majority of early (78.6%) and mid to post pubertal (77.1%) girls chose silhouettes (3–4) which represented a normal BMI. When dividing the girls according to their BMI category, among overweight/obese participants, 11.4% of early pubertal chose silhouettes (3–5) and 19.5% of mid to post pubertal girls selected silhouettes (4–5), representing “normal and overweight” BMI category as their feel body image. Among underweight participants, 10% of early pubertal girls chose silhouettes (4–5) representing “normal and overweight” BMI category and 3.9% of mid to post pubertal girls chose normal silhouettes (3–4) as their ideal body image (Table 3).

**Table 3:** Characteristics of Stunkard’s silhouettes chosen by early and mid to post pubertal girls to represent different dimensions of body image.

**Silhouettes**	**Early vs. Mid to Post-Pubertal Girls (Mean±sd)**			**P-value**
**Feel body image**	3.0±1.6 vs. 3.5±1.4			0.331
**Ideal body image**	4.3±1.8 vs. 4.1±1.5			**0.045**
**FID scores**	1.3±1.9 vs. 0.5±1.8			0.244
	**BMI Categories**			
	**Early vs. Mid to Post-Pubertal Girls (Mean±sd)**			
**Silhouettes**	**Underweight**	**Normal weight**	**Overweight & obesed**	**P-value**
**Feel body image**	1.9±1.5 vs. 2.5±0.6	3.0±1.5 vs. 3.3±1.3	4.1±1.5 vs. 4.5±1.5	0.669
**Ideal body image**	4.8±1.9 vs. 3.5±0.6	4.3±1.9 vs. 4.1±1.4	3.8±1.0 vs. 4.0±1.5	0.637
**FID scores**	3.0±1.0 vs. 1.0±0.8	1.2±1.9 vs. 0.7±1.6	0.4±1.8 vs. −0.5±2.0	0.399

Values are presented as means ± standard deviation (SD) using ANOVA (one-way).

Differences in EAT-26 total scores and EAT-26 sub-components (dieting, bulimia and food preoccupation and oral control) between the sexes and between pubertal groups were not significant, however the early pubertal girls had significantly higher mean value for oral control than boys of the same pubertal stage (4.7 vs. 3.4, p = 0.024).

The silhouettes chosen to represent various attributes were consistent between the early and mid to post pubertal girls (Table 4). The majority of girls in both pubertal groups associated the overweight/obese silhouettes with being the worst, having more respect, having less respect, being the strongest, and the happiest. In contrast, underweight silhouettes were associated with only negative attributes—being clumsy, being weak and being unhappy. Most of the girls in both pubertal groups considered the normal silhouettes to be the “best”. There was a significant difference between the pubertal groups with regard to the silhouettes which best represented the “weakest” (p = 0.010) and “unhappiest” (p = 0.030).

**Table 4:** Female body silhouettes with attributes as assessed by early and mid to post pubertal girls.

Silhouettes	Early-Pubertal Girls	Mid to Post-Pubertal Girls	P-value
**Best**			
Underweight	19.0%	14.5%	
Normal	50.0%	60.0%	0.471
Overweight and obesed combined	31.0%	25.5%	
**Worst**			
Underweight	31.0%	29.0%	
Normal	0.0%	1.0%	0.963
Overweight and obesed combined	69.0%	70.0%	
**Clumsy**			
Underweight	54.4%	52.1%	
Normal	4.4%	1.7%	0.466
Overweight and obesed combined	41.2%	46.2%	
**More respect**			
Underweight	20.5%	16.2%	
Normal	38.1%	34.2%	0.590
Overweight and obesed combined	41.4%	49.6%	
**Less respect**			
Underweight	26.5%	36.7%	
Normal	7.4%	14.5%	0.134
Overweight and obesed combined	66.1%	47.8%	
**Strongest**			
Underweight	4.4%	6.7%	
Normal	10.3%	6.0%	0.458
Overweight and obesed combined	85.3%	86.3%	
**Weakest**			
Underweight	79.4%	87.2%	
Normal	9.0%	0.0%	**0.010 χ^2^ = 10.870**
Overweight and obesed combined	11.6%	12.8%	
**Happiest**			
Underweight	16.2%	14.5%	
Normal	36.6%	36.7%	0.849
Overweight and obesed combined	47.2%	48.8%	
**Unhappiest**			
Underweight	84.0%	85.5%	
Normal	0.0%	6.0%	0.030 **χ^2^ = 8.444**
Overweight and obesed combined	16.0%	8.5%	

Comparison by pubertal stage was computed using *χ*^2^-tests and (%) are reported.

The silhouettes chosen to represent various attributes were also consistent between the early and mid to post pubertal boys (Table 5). The majority of boys in both pubertal groups associated the overweight and obese silhouettes with being the “worst”, having “more respect”, having “less respect”, being the “strongest”, and the “happiest”. The majority of boys in both pubertal groups associated the negative attributes “weakest” and “unhappy” with the underweight silhouettes. There was a significant difference between the pubertal groups with regard to the silhouette that represented an “unhappy” attribute (p = 0.020).

**Table 5:** Female body silhouettes with attributes as assessed by early and mid to post pubertal boys.

Silhouettes	Early-Pubertal Boys	Mid to Post-Pubertal Boys	P-value
**Best**			
Underweight	10.5%	3.3%	
Normal	43.2%	53.3%	0.087
Overweight and obesed combined	46.3%	43.4%	
**Worst**			
Underweight	48.4%	40.0%	
Normal	1.1%	0.0%	0.266
Overweight and obesed combined	50.5%	60.0%	
**Clumsy**			
Underweight	45.3%	53.3%	
Normal	8.3%	3.3%	0.436
Overweight and obesed combined	46.4%	43.4%	
**More respect**			
Underweight	6.3%	15.6%	
Normal	34.7%	25.6%	0.175
Overweight and obesed combined	59.0%	58.8%	
**Less respect**			
Underweight	35.8%	35.6%	
Normal	9.5%	7.7%	0.918
Overweight and obesed combined	54.7%	56.7%	
**Strongest**			
Underweight	6.3%	3.3%	
Normal	6.3%	6.6%	0.798
Overweight and obesed combined	87.4%	90.1%	
**Weakest**			
Underweight	83.2%	90.0%	
Normal	7.3%	2.2%	0.228
Overweight and obesed combined	9.5%	7.8%	
**Happiest**			
Underweight	9.5%	10.0%	
Normal	35.8%	36.7%	0.929
Overweight and obesed combined	54.7%	53.3%	
**Unhappiest**			
Underweight	91.5%	90.0%	
Normal	1.1%	0.0%	**0.020 χ^2^ = 8.085**
Overweight and obesed combined	7.4%	10.0%	

Comparison by pubertal stage was computed using *χ*^2^-tests and (%) are reported.

## Discussion

To our knowledge, this is the first study reporting on the associations between BMI, disordered eating attitude, body dissatisfaction in female adolescents, and descriptive attributes assigned to silhouettes of varying sizes in male and female adolescents from rural South Africa. In this study, the prevalence of overweight and obesity was higher in girls than boys (16.4% vs 2.6%), and was greater in late puberty compared to early puberty. The prevalence of overweight and obesity that we report in this sample of rural adolescents was lower than the 2008 National Youth Risk Behaviour Survey [31], but consistent with other studies [32, 33]. In contrast, a 2012 study, conducted on rural school children (aged between 10 and 16 years) reported that although the prevalence of overweight was higher in girls than boys, obesity was more prevalent in boys [23]. In that study, 11% of the girls and 9% of the boys were overweight, while 5.5% and 4.4% of boys and girls were obese, respectively. Therefore, the focus of interventions within the public health context should be improved through school-based nutrition and physical activity programmes among South African children and youth [34].

Our study also showed a consistent trend, where early and mid to post pubertal girls who desired to be fatter had the highest body mass index and those who desired to be thinner had the lowest body mass index in their respective pubertal groups. We found that the majority (83.5%) of the girls demonstrated body dissatisfaction, with 58% expressing a desire for a thinner body size. Interestingly, with regard to BMI categories which represented the silhouettes chosen by the girls to represent themselves, the overweight/obese girls chose silhouettes with a lower BMI than their actual BMI. Further, girls in the underweight and normal weight groups expressed a desire to be thinner (positive FID scores).

Recent evidence from South Africa has shown that many urban children and adolescents are less satisfied with their body shape [5, 17, 18] Similarly, the majority of both our early and mid to post pubertal girls wanted to be thinner, with the proportion of those who preferred to be thinner being higher in early rather than mid to post pubertal girls (61.4% vs. 56%). We have shown that BMI was higher in participants who desired to be fatter, compared to the BMI of the girls who wished to be thinner. Similar to earlier South African studies [7, 8, 19], 48.9% of the the girls with a normal BMI wished to be thin in the present study. In a study by Gitau et al., (2014), the majority (59.6%) of urban Black and White girls, aged between 13 and 17 years wanted to be thin, whilst 38.8% of Black girls had higher body dissatisfaction compared to White girls (16.7%) [19].

In another study by Puoane et al., (2013), 63% of Black schoolgirls aged between 10 and 19 years, living in poor suburbs of Cape Town, had a desire for a low BMI (<21.7) [7]. Our results also mirror findings from developed and other low and middle income countries [6, 13, 35, 36] countries, Ricciardelli and McCabe (2001), showed that the desire for thinner body size amongst girls from different countries ranges from 28% to 55% [13]. Of concern is that 5.8% of the underweight girls expressed a desire to be thinner. This may lead to food restriction and excessive exercise to change their bodies, which may place them at a greater health risk and lead to pathological eating disorders such as anorexia nervosa and bulimia nervosa [37].

Nearly 20% of the mid to post pubertal girls, who were classified as overweight/obese wanted to be fatter. Similarly, studies have also reported a trend towards a larger figure in Black populations and evidence suggests that being overweight is traditionally accepted in African culture as a sign of wealth, beauty and the absence of illness [15–17]. This is of concern given the health consequences of obesity and that almost 80% of overweight children become overweight and obese adults [38]. Therefore, primary prevention of obesity should start at an early age, particularly for girls [39].

Our study contributes to the body of literature on the risk for future eating disorder in South African adolescents. The prevalence rates of EAT-26 score ≥ 20 in this study (10.6% and 8% for early and mid to post pubertal girls, respectively) were higher than previously reported a decade ago (3%) for older (17.8 years) rural South African female adolescents [20]. In comparison, this was lower than 13% reported for young students (aged 20 years) from rural and urban origins, from the University of the North, Limpopo Province [21]. Beyond the rural settings, the percentages of abnormal eating attitudes found in the present study were within range of studies conducted in Johannesburg on urban Black adolescent girls aged 11 years (1%) and among adolescents between 13 and 17 years old (11%, and 13.1%) respectively [5, 11]. The only other study on South African male subjects showed that more urban Black than White males (40.2% vs. 5.2%) (aged 13, 15 and 17 years) had abnormal eating attitudes [18]. These prevalences are much higher than the 10.5% and 7.1% of our early and mid to post pubertal boys in the current study, indicating important transitional difference between the two settings.

These findings are the first to describe attributes assigned to silhouettes of varying sizes, amongst male and female adolescents in rural South Africa, but also shows conflicting perceptions as the majority of the adolescents still associated the overweight/obese silhouette with positive attributes including more respect, strongest, and happiest, and the underweight silhouette with only negative attributes (weak and unhappy), which might be associated with illness such as HIV/AIDS and tuberculosis [40].

The strength of this study is the inclusion of an under-studied geographical population whose particular health needs have been under-served and poorly delineated in the past. In addition, the questionnaires used in this study were validated internationally and within South Africa and the interview techniques were carried out by experienced fieldworkers who followed standardised procedures used in the Birth to Twenty longitudinal cohort.

This study had limitations, including the low internal consistency for EAT-26 and EAT-26 sub-components and therefore these results should be interpreted with caution. Examination revealed that some question items may have been poorly understood despite the questions being translated into the local language. It is possible that in a rural setting where food insecurity is still prevalent, some of the concepts around not eating, vomiting or dieting practices, are less well understood or engaged in. This suggests a need for critical research around both the methodological value of the EAT-26 and its applicability in rural South Africa and how food insecurity impacts understanding of certain eating practices. Second, the cross-sectional design of this study does not allow for cause-effect relationship. Third, the results can not be generalised to other rural settings or population groups because of the fact the sample included adolescents aged between 11 to 15 years. Another study limitation is the recall bias and the motivation of the participants in the interview setting [41]. Nevertheless, these findings from this rural setting, in conjunction with previous studies in South Africa, may be useful to guide future research interventions.

## Conclusions

In summary, the findings of this study confirmed the higher prevalence of combined overweight and obesity in females than males. Our results highlighted that the majority of rural adolescent girls are dissatisfied with their body sizes, and this differs by BMI category, but indicates growing desire for Western ideals of thinness. This is most likely associated with the socio-economic transition that has taken place in South Africa within the last two decades and greater exposure to “Western” media. The study findings suggest that many adolescents are struggling with body image and desiring “unhealthy” body shapes, either too thin or too fat. This necessitates the critical need for policy intervention which would aim to educate and promote a healthy body size in adolescence and improve access to adolescent counselling support around eating disorder pathology and body image dissatisfaction.

## Supporting Information

S1 File This is the raw data collected and analysed for this study. Analysis of: (a) BMI, (b) disordered eating attitudes, (c) female adolescents’ body dissatisfaction, and (d) male and female silhouettes’ descriptive attributes.
(DTA)
